# Fostering Creativity and Critical Thinking in College: A Cross-Cultural Investigation

**DOI:** 10.3389/fpsyg.2021.760351

**Published:** 2021-11-11

**Authors:** Ji Hoon Park, Weihua Niu, Li Cheng, Heavon Allen

**Affiliations:** ^1^Department of Psychology, Pace University, New York, NY, United States; ^2^Developmental and Educational Research Center for Children's Creativity, Faculty of Education, Beijing Normal University, Beijing, China

**Keywords:** creativity, critical thinking, cross-cultural differences, college, research experience

## Abstract

Enhancing creativity and critical thinking have garnered the attention of educators and researchers for decades. They have been highlighted as essential skills for the 21st century. A total of 103 United States students (53 female, 24 male, two non-binary, and 24 non-reporting) and 166 Chinese students (128 female, 30 male, one non-binary, and seven non-reporting) completed an online survey. The survey includes the STEAM-related creative problem solving, Sternberg scientific reasoning tasks, psychological critical thinking (PCT) exam, California critical thinking (CCT) skills test, and college experience survey, as well as a demographic questionnaire. A confirmatory factor analysis (CFA) yields a two-factor model for all creativity and critical thinking measurements. Yet, the two latent factors are strongly associated with each other (*r*=0.84). Moreover, Chinese students outperform American students in measures of critical thinking, whereas Americans outperform Chinese students in measures of creativity. Lastly, the results also demonstrate that having some college research experience (such as taking research method courses) could positively influence both United States and Chinese students’ creativity and critical thinking skills. Implications are discussed.

## Introduction

Creativity and critical thinking have been recognized as essential skills in the 21st century (National Education Association, 2012). Many researchers and educators have focused on these two skills, including acquisition, enhancement, and performance. In addition, numerous studies have been devoted to understanding the conceptual complexities involved in creativity and critical thinking. Although similar to each other, creativity and critical thinking are distinctive by definition, each with a different emphasis.

The concept of creativity has evolved over the years. It was almost exclusively conceptualized as divergent thinking when Guilford (1956, 1986) proposed divergent thinking as a part of intelligence. Earlier measures of creativity took the approach of divergent thinking, measuring creative potential (Wallach and Kogan, 1965; Torrance, 1966, 1988; Runco and Albert, 1986; Kim, 2005). In 1990s, many creativity scholars challenged the validity of tests of divergent thinking, and suggested that divergent thinking only captures the trivial sense of creativity, and proposed to use the product-oriented method to measure creativity (Csikszentmihalyi, 1988; Amabile, 1996; Sternberg and Lubart, 1999). A system model of creativity, which recognizes the important roles individual, field, and domain have played, was used as a framework to conceptualize creativity. A widely accepted definition for creativity is a person’s ability to generate an idea or product that is deemed as both novel and appropriate by experts in a field of human activities (Scott and Bruce, 1994; Amabile, 1996; Csikszentmihalyi, 1999; Sternberg and Lubart, 1999; Hunter et al., 2007). Corazza and Lubart (2021) recently proposed a dynamic definition of creativity, in which creativity is defined as a context-embedded phenomenon that is tightly related to the cultural and social environment. Based on this new definition, measures of creativity should be context-specific and culturally relevant, especially when it is examined cross-culturally.

Similarly, the conceptualization of critical thinking has also evolved over the years. Earlier definitions emphasized the broad multidimensional aspects of critical thinking, including at least three aspects: attitude, knowledge, and skills (Glaser, 1941). The definition has been evolved to include specific components for each aspect (Watson and Glaser, 1980). For example, critical thinking is recognized as the ability to use cognitive skills or strategies to increase the probability of a desirable outcome (Halpern, 1999). More specifically, cognitive skills such as evaluation, problem-solving, reflective thinking, logical reasoning, and probability thinking are recognized as parts of critical thinking skills in research and assessments (Ennis, 1987, Scriven and Paul, 1987, Halpern, 1999). Moving into the 21st century, metacognition and self-regulatory skills have also become essential components for critical thinking in addition to the cognitive skills recognized by earlier scholars (Korn, 2014, Paul and Elder, 2019).

Similar to the concept of creativity, critical thinking is also viewed as multidimensional and domain specific (Bensley and Murtagh, 2012). For example, critical thinking in psychology, also referred to as psychological critical thinking (PCT), is defined as one’s ability to evaluate claims in a way that explicitly incorporates basic principles of psychological science (Lawson, 1999). As one of the important hub sciences, psychology is often regarded as a foundational course for scientific training in American higher education (Boyack et al., 2005). In psychological discourse, critical thinking is often defined in tandem with scientific thinking, which places significance on hypothesis-testing and problem-solving in order to reduce bias and erroneous beliefs (Halpern, 1984; American Psychological Association, 2016; Lamont, 2020; Sternberg and Halpern, 2020). Based on this definition, measures of critical thinking should assess cognitive skills (i.e., evaluation, logical reasoning) and ability to utilize scientific methods for problem-solving.

In addition to the evolution of the definitions of critical thinking and creativity, research into these two concepts has led to the development of various measurements. For both concepts, there have been numerous measurements that have been studied, utilized, and improved.

The complexities associated with creativity (i.e., context-relevant and domain-specificity) pose a major issue for its measurement. Many different types of creativity measures have been developed in the past. Measures using a divergent thinking approach, such as the Torrance Tests of Creative Thinking (Torrance, 1974) and Alternate Uses Test (Guilford et al., 1960), a product-oriented approach, a third person nomination approach, as well as a self-report approach measuring personality (Gough, 1979), creative behavior (Hocevar and Michael, 1979; Rodriguez-Boerwinkle et al., 2021), and creative achievement (Carson et al., 2005; Diedrich et al., 2018).

Both the divergent thinking and the product-oriented approaches have been widely used in the creativity literature to objectively measure creativity. The tasks of both approaches are generally heuristic, meaning that no correct answer is expected and the process does not need to be rational. When scoring divergent thinking, the number of responses (i.e., fluency) and the rareness of the response (i.e., originality) were used to represent creativity. When scoring products using the product-orientated approach, a group of experts provides their subjective ratings on various dimensions such as originality, appropriateness, and aesthetically appealing to these products using their subjective criteria. When there is a consensus among the experts, average ratings of these expert scores are used to represent the creativity of the products. This approach is also named as Consensual Assessment Technique (CAT; Amabile, 1982, 1996). Some scholars viewed the CAT approach as focusing on the convergent aspect of creativity (Lubart et al., 2013). Recognizing the importance of divergent and convergent thinking in conceptualizing creativity, Lubart et al. (2013) have suggested including divergent thinking and product-oriented approach (i.e., CAT) to objective measures of creativity (Barbot et al., 2011).

Similar to measures of creativity, measurements of critical thinking are also multilevel and multi-approach. In an article reviewing the construction of critical thinking in psychological studies, Lamont (2020) argues that critical thinking became a scientific object when psychologists attempted to measure it. Different from measures of creativity, where the tasks are heuristic in nature, measures of critical thinking require participants to engage in logical thinking. Therefore, the nature of critical thinking tasks is more algorithmic.

The interest in the study of critical thinking is evident in the increased efforts in the past decades to measure such a complex, multidimensional skill. Watson-Glaser Tests for Critical Thinking (Watson and Glaser, 1938) is widely recognized as the first official measure of critical thinking. Since then, numerous measurements of critical thinking have been developed to evaluate both overall and domain-specific critical thinking, such as the PCT Exam (Lawson, 1999; See Mueller et al., 2020 for list of assessments). A few of the most commonly used contemporary measures of critical thinking include the Watson-Glaser Test for Critical Thinking Appraisals (Watson and Glaser, 1980), Cornell Critical Thinking Test (Ennis et al., 1985), and California Critical Thinking (CCT) Skills Test (Facione and Facione, 1994). As the best established and widely used standardized critical thinking measures, these tests have been validated in various studies and have been used as a criterion for meta-analyses (Niu et al., 2013; Ross et al., 2013).

There have also been concerns regarding the usage of these standardized measures of critical thinking on its own due to its emphasis on measuring general cognitive abilities of participants, while negating the domain-specific aspect of critical thinking (Lamont, 2020). The issues associated with standardized measures are not unique to standardized critical thinking measures, as same types of criticisms have been raised for standardized college admissions measures such as the Graduate Record Exam (GRE). To develop an assessment that encompasses a broader range of student abilities that is more aligned to scientific disciplines, Sternberg and Sternberg (2017) developed a scientific inquiry and reasoning measure. This measure is aimed to assess participants’ ability to utilize scientific methods and to think scientifically in order to investigate a topic or solve a problem (Sternberg and Sternberg, 2017). The strength of this measure is that it assesses students’ abilities (i.e., ability to think critically) that are domain-specific and relevant to the sciences. Considering the multidimensional aspect of critical thinking, a combination of a standardized critical thinking measure, an assessment measuring cognitive abilities involved in critical thinking; and a measure that assesses domain-specific critical thinking, would provide a comprehensive evaluation of critical thinking.

### The Relationship Between Creativity and Critical Thinking

Most of the studies thus far referenced have investigated creativity and critical thinking separately; however, the discussion on the relationship between creativity and critical thinking spans decades of research (Barron and Harrington, 1981; Glassner and Schwartz, 2007; Wechsler et al., 2018; Akpur, 2020). Some earlier studies on the relationship between divergent thinking and critical thinking have observed a moderate correlation (*r*=0.23, *p*<0.05) between the two (Gibson et al., 1968). Using measures of creative personality, Gadzella and Penland (1995) also found a moderate correlation (*r*=0.36, *p*<0.05) between creative personality and critical thinking.

Recent studies have further supported the positive correlation between critical thinking and creativity. For example, using the creative thinking disposition scale to measure creativity, Akpur (2020) found a moderate correlation between the two among college students (*r*=0.27, *p*<0.05). Similarly, using the critical thinking disposition scale to measure critical thinking and scientific creativity scale and creative self-efficacy scale to measure creativity, Qiang et al. (2020) studied the relationship between critical thinking and creativity to a large sample of high school students (*n*=1,153). They found that the relationship between the two varied depending on the type of measurement of creativity. More specifically, the correlation between critical thinking disposition and creative self-efficacy was *r*=0.045 (*p*<0.001), whereas the correlation between critical thinking disposition and scientific creativity was *r*=0.15 (*p*<0.01).

Recognizing the moderate relationship between the two, researchers have also aimed to study the independence of creativity and critical thinking. Some studies have found evidence that these constructs are relatively autonomous. The results of Wechsler et al. (2018) study, which aimed to investigate whether creativity and critical thinking are independent or complementary processes, found a relative autonomy of creativity and critical thinking and found that the variables were only moderately correlated. The researchers in this study suggest that a model that differentiated the two latent variables associated with creativity and critical thinking dimensions was the most appropriate method of analysis (Wechsler et al., 2018). Evidence to suggest that creativity and critical thinking are fairly independent processes was also found in study of Ling and Loh (2020). The results of their research, which examined the relationship of creativity and critical thinking to pattern recognition, revealed that creativity is a weak predictor of pattern recognition. In contrast, critical thinking is a good predictor (Ling and Loh, 2020).

It is worth noting that a possible explanation for the inconsistencies in these studies’ results is the variance in the definition and the measures used to evaluate creativity and critical thinking. Based on the current literature on the relationship between creativity and critical thinking, we believe that more investigation was needed to further clarify the relationship between creativity and critical thinking which became a catalyst for the current study.

### Cross-Cultural Differences in Creativity and Critical Thinking Performance

Results from various cross-cultural studies suggest that there are differences in creativity and critical thinking skills among cultures. A common belief is that individuals from Western cultures are believed to be more critical and creative compared to non-Westerners, whereas individuals from non-Western cultures are believed to be better at critical thinking related tasks compared to Westerners (Ng, 2001; Wong and Niu, 2013; Lee et al., 2015). For example, Wong and Niu (2013) found a persistent cultural stereotype regarding creativity and critical thinking skills that exist cross-culturally. In their study, both Chinese and Americans believed that Chinese perform better in deductive reasoning (a skill comparable to critical thinking) and that Americans perform better on creativity. This stereotype belief was found to be incredibly persistent as participants did not change their opinions even when presented with data that contradicted their beliefs.

Interestingly, research does suggest that such a stereotype might be based on scientific evidence (Niu et al., 2007; Wong and Niu, 2013). In the same study, it was revealed that Chinese did in fact perform better than Americans in deductive reasoning, and Americans performed better in creativity tests (Wong and Niu, 2013). Similarly, Lee et al. (2015) found that compared to American students, Korean students believed that they are more prone to use receptive learning abilities (remembering and reproducing what is taught) instead of critical and creative learning abilities.

### Cultural Influence on Critical Thinking

Other studies investigating the cultural influence on critical thinking have had more nuanced findings. Manalo et al. (2013) study of university students from New Zealand and Japan found that culture-related factors (self-construal, regulatory mode, and self-efficacy) do influence students’ critical thinking use. Still, the differences in those factors do not necessarily equate to differences in critical thinking. Their results found that students from Western and Asian cultural environments did not have significant differences in their reported use of critical thinking. The researchers in this study suggest that perhaps the skills and values nurtured in the educational environment have a more significant influence on students’ use of critical thinking (Manalo et al., 2013).

Another study found that New Zealand European students performed better on objective measures of critical thinking than Chinese students. Still, such differences could be explained by the student’s English proficiency and not dialectical thinking style. It was also revealed in this study that Chinese students tended to rely more on dialectical thinking to solve critical thinking problems compared to the New Zealand European students (Lun et al., 2010). Other research on the cultural differences in thinking styles revealed that Westerners are more likely to use formal logical rules in reasoning. In contrast, Asians are more likely to use intuitive experience-based sense when solving critical thinking problems (Nisbett et al., 2001).

These studies suggest that culture can be used as a broad taxonomy to explain differences in critical thinking use. Still, one must consider the educational environment and thinking styles when studying the nature of the observed discrepancies. For instance, cultural differences in thinking style, in particular, might explain why Westerners perform better on some critical thinking measures, whereas Easterners perform better on others.

### Cultural Influence on Creative Performance

Historically, creativity studies have suggested that individuals from non-Western cultures are not as creative as Westerners (Torrance, 1974; Jellen and Urban, 1989; Niu and Sternberg, 2001; Tang et al., 2015). For example, in one study, Americans generated more aesthetically pleasing artworks (as judged by both American and Chinese judges) than Chinese (Niu and Sternberg, 2001). However, recent creativity research has suggested that cross-cultural differences are primarily attributable to the definition of creativity rather than the level of creativity between cultures. As aforementioned, creativity is defined as an idea or product that is both novel and appropriate. Many cross-cultural studies have found that Westerners have a preference and perform better in the novelty aspect, and Easterners have a preference and perform better in the appropriateness aspect. In cross-cultural studies, Rockstuhl and Ng (2008) found that Israelis tend to generate more original ideas than their Singaporean counterparts. In contrast, Singaporeans tend to produce more appropriate ideas. Bechtoldt et al. (2012) found in their study that Koreans generated more useful ideas, whereas Dutch students developed more original ideas. Liou and Lan (2018) found Taiwanese tend to create and select more useful ideas, whereas Americans tend to generate and choose more novel ideas. The differences in creativity preference and performance found in these studies suggest that cultural influence is a prominent factor in creativity.

In summary, cross-cultural studies have supported the notion that culture influences both creativity and critical thinking. This cultural influence seems relatively unambiguous in creativity as it has been found in multiple studies that cultural background can explain differences in performance and preference to the dual features of creativity. Critical thinking has also been influenced by culture, albeit in an opaquer nature in comparison to creativity. Critical thinking is ubiquitous in all cultures, but the conception of critical thinking and the methods used to think critically (i.e., thinking styles) are influenced by cultural factors.

### Influence of College Experience on Creativity and Critical Thinking

Given its significance as a core academic ability, the hypothesis of many colleges and universities emphasize that students will gain critical thinking skills as the result of their education. Fortunately, studies have shown that these efforts have had some promising outcomes. Around 92% of students in multi-institution research reported gains in critical thinking. Only 8.9% of students believed that their critical thinking had not changed or had grown weaker (Tsui, 1998). A more recent meta-analysis by Huber and Kuncel (2016) found that students make substantial gains in critical thinking during college. In addition, the efforts to enhance necessary thinking skills have led to the development of various skill-specific courses. Mill et al. (1994) found that among three groups of undergraduate students, a group that received tutorial sessions and took research methodology and statistics performed significantly better on scientific reasoning and critical thinking abilities tests than control groups. Penningroth et al. (2007) found that students who took a class in which they were required to engage in active learning and critical evaluation of claims by applying scientific concepts, had greater improvement in psychological critical thinking than students in the comparison groups. There have also been studies in which students’ scientific inquiry and critical thinking skills have improved by taking a course designed with specific science thinking and reasoning modules (Stevens and Witkow, 2014; Stevens et al., 2016).

Using a Survey of Undergraduate Research Experience (SURE), Lopatto (2004, 2008) found that research experience can help students gain various learning skills such as ability to integrate theory and practice, ability to analyze data, skill in the interpretation of results, and understanding how scientists work on problem. All of these learning skills correspond to at least one of the dimensions mentioned earlier in the definition of critical thinking (i.e., evaluation, analytical thinking, and problem solving through). Thus, results of SURE provide evidence that critical thinking can be enhanced through research experience (Lopatto, 2004, 2008).

In comparison to critical thinking, only a few studies have examined the interaction between creativity and college experience. Previous research on STEM provides some evidence to suggest that STEM education can promote the learner’s creativity (Land, 2013, Guo and Woulfin, 2016, Kuo et al., 2018). Notably, study of Kuo et al. (2018) suggest that project-based learning in STEM has the merits of improving one’s creativity. They found that the STEM Interdisciplinary Project-Based Learning (IPBL) course is a practical approach to improve college student’s creativity (Kuo et al., 2018). College research experience in particular, has been reported as important or very important by faculty and students for learning how to approach problems creatively (Zydney et al., 2002).

Although specific college courses aimed to enhance creativity have been scarce, some training programs have been developed specifically to improve creativity. Scott et al. (2004) conducted a quantitative review of various creativity training and found that divergent thinking, creative problem solving, and creativity performance can be enhanced through skill-specific training programs. Embodied creativity training programs, consisting of creativity fitness exercises and intensive workshops, have also been effective in enhancing participants’ creative production and improving their creative self-efficacy (Byrge and Tang, 2015).

Both critical thinking and creativity were also found to be important in students’ learning. Using a longitudinal design for one semester to 52 graduate students in biology, Siburian et al. (2019) studied how critical thinking and creative thinking contribute to improving cognitive learning skills. They found that both critical and creative thinking significantly contributes to enhancing cognitive learning skills (*R*^2^=0.728). They each contribute separately to the development of cognitive learning skills (*b* was 0.123 between critical thinking and cognitive learning and 0.765 between creative thinking and cognitive learning). The results from research on creativity and critical thinking indicate that training and experiences of students in college can enhance both of these skills.

## Current Study

Previous literature on creativity and critical thinking suggests that there is a positive correlation between these two skills. Moreover, cultural background influences creativity and critical thinking conception and performance. However, our literature review suggests that there are only a few studies that have investigated creativity and critical thinking simultaneously to examine whether cultural background is a significant influence in performance. In addition, most of the past research on creativity and critical thinking have relied on dispositions or self-reports to measure the two skills and the investigation on the actual performance have been scarce. Lastly, past studies suggest that the acquisition and enhancement of these skills are influenced by various factors. Notably, college experience and skill-specific training have been found to improve both creativity and critical thinking. However, it is not yet clear how college experience aids in fostering creativity and critical thinking and which elements of college education are beneficial for enhancing these two skills. The cultural influence on creativity and critical thinking performance also needs further investigation.

The current study aimed to answer two questions related to this line of thought. How does culture influence creativity and critical thinking performance? How does college experience affect creativity and critical thinking? Based on past findings, we developed three hypotheses. First, we hypothesized that there is a positive association between critical thinking and creativity. Second, we suggest that college students from different countries have different levels of creativity and critical thinking. More specifically, we predicted that United States students would perform better than Chinese students on both creativity and critical thinking. Last, we hypothesized that having college research experience (through courses or research labs) will enhance creativity and critical thinking.

## Materials and Methods

### Participants

The study was examined by the Internal Review Board by the host university in the United States and obtained an agreement from a partner university in China to meet the ethical standard of both countries.

Participants include 103 university students from the United States and 166 university students from Mainland China. Among all participants, 181 were female (67.3%), 54 were male (20.1%), non-binary or gender fluid (*n*=3, 1.1%), and some did not report their gender (*n*=31, 11.5%). The majority of participants majored in social sciences (*n*=197, 73.2%). Other disciplines include business and management (*n*=38, 14.1%), engineering and IT (*n*=20, 7.4%), and sciences (*n*=14, 5.2%). A Chi-square analysis was performed to see if the background in major was different between the American and Chinese samples. The results showed that the two samples are comparable in college majors, *X*^2^_(3, 265)_=5.50, *p*=0.138.

The American participants were recruited through campus recruitment flyers and a commercial website called Prolific (online survey distribution website). Ethnicities of the American participants were White (*n*=44, 42.7%), Asian (*n*=13, 12.6%), Black or African American (*n*=11, 10.7%), Hispanic or Latinos (*n*=5, 4.9%), and some did not report their ethnicity (*n*=30, 29.1%). The Chinese participants were recruited through online recruitment flyers. All Chinese students were of Han ethnicity.

After reviewing and signing an online consent form, both samples completed a Qualtrics survey containing creativity and critical thinking measures.

### Measurements

#### STEAM Related Creative Problem Solving

This is a self-designed measurement, examining participant’s divergent and convergent creative thinking in solving STEAM-related real-life problems. It includes three vignettes, each depicting an issue that needs to be resolved. Participants were given a choice to pick two vignettes to which they would like to provide possible solutions for. Participants were asked to provide their answers in two parts. In the first part, participants were asked to provide as many solutions as they can think of for the problem depicted (divergent). In the second part, participants were asked to choose one of the solutions they gave in the first part that they believe is the most creative and elaborate on how they would carry out the solution (convergent).

The responses for the first part of the problem (i.e., divergent) were scored based on fluency (number of solutions given). Each participant received a score on fluency by averaging the number of solutions given across three tasks. In order to score the originality of the second part of the solution (i.e., convergent), we invited four graduate students who studied creativity for at least 1year as expert judges to independently rate the originality of all solutions. The Cronbach’s Alpha of the expert ratings was acceptable for all three vignette solutions (0.809, 0.906, and 0.703). We then averaged the originality scores provided by the four experts to represent the originality of each solution. We then averaged the top three solutions as rated by the experts to represent the student’s performance on originality. In the end, each student received two scores on this task: fluency and originality.

#### Psychological Critical Thinking Exam

We adopted an updated PCT Exam developed by Lawson et al. (2015), which made improvements to the original measure (Lawson, 1999). We used PCT to measure the participants’ domain-specific critical thinking: critical thinking involved in the sciences. The initial assessment aimed to examine the critical thinking of psychology majors; however, the updated measure was developed so that it can be used to examine students’ critical thinking in a variety of majors. The split-half reliability of the revised measurement was 0.88, and test-retest reliability was 0.90 (Lawson et al., 2015). Participants were asked to identify issues with a problematic claim made in two short vignettes. For example, one of the questions states:

*Over the past few years, Jody has had several dreams that apparently predicted actual events. For example, in one dream, she saw a car accident and later that week she saw a van run into the side of a pickup truck. In another dream, she saw dark black clouds and lightning and 2days later a loud thunderstorm hit her neighborhood. She believes these events are evidence that she has a psychic ability to predict the future through her dreams. Could the event have occurred by chance? State whether or not there is a problem with the person’s conclusions and explain the problem (if there is one).*

Responses were scored based on the rubric provided in the original measurement (Lawson et al., 2015). If no problem was identified the participants would receive zero points. If a problem was recognized but misidentified, the participants would receive one point. If the main problem was identified and other less relevant problems were identified, the participants received two points. If participants identified only the main problem, they received three points. Following the rubric, four graduate students independently rated the students’ critical thinking task. The Cronbach’s Alpha of the expert ratings was acceptable for both vignettes (0.773 and 0.712). The average of the four scores given by the experts was used as the final score for the participants.

#### California Critical Thinking Skills Test

This objective measure of critical thinking was developed by Facione and Facione (1994). We used CCT to measure a few of the multidimensions of critical thinking such as evaluation, logical reasoning, and probability thinking. Five sample items provided from Insight Assessment were used instead of the standard 40-min long CCT. Participants were presented with everyday scenarios with 4–6 answer choices. Participants were asked to make an accurate and complete interpretation of the question in order to correctly answer the question by choosing the right answer choice (each correct answer was worth one point). This test is commonly used to measure critical thinking, and previous research has reported its reliability as *r*=0.86 (Hariri and Bagherinejad, 2012).

#### Sternberg Scientific Inquiry and Reasoning

This measure was developed by Sternberg and Sternberg (2017) as an assessment of scientific reasoning. We used this assessment as a domain-specific assessment to measure participants’ scientific creativity (generating testable hypotheses) and scientific critical thinking involved in generating experiments. For this two-part measure, participants were asked to read two short vignettes. For one of the vignettes, participants were asked to generate as many hypotheses as possible to explain the events described in the vignette. For the other, create an experiment to test the hypothesis mentioned in the vignette.

After carefully reviewing the measurement, we notice that the nature of the tasks in the first part of this measure (hypothesis generation) relied on heuristics, requiring participants to engage in divergent thinking. The number of valid hypotheses provided (i.e., fluency) was used to represent the performance of this task. We, therefore, deem that this part measures creativity. In contrast, the second part of the measure, experiment generation, asked participants to use valid scientific methods to design an experiment following the procedure of critical thinking such as evaluation, problem-solving, and task evaluation. Its scoring also followed algorithms so that a correct answer could be achieved. For the above reasons, we believe hypotheses generation is a measurement of creativity and experiment generation is a measurement for critical thinking.

Based on the recommended scoring manual, one graduate student calculated the fluency score from the hypothesis generation measurement. Four experts read through all students’ responses to the experiment generation. They discussed a rubric on how to score these responses, using a four-point scale, with a “0” representing no response or wrong response, a “1” representing partially correct, a “2” representing correct response. An additional point (the three points) was added if the participant provided multiple design methods. Based on the above rubric, the four experts independently scored this part of the questionnaire. The Cronbach’s Alpha of the four expert ratings was 0.792. The average score of the four judges was used to represent their critical thinking scores on this task.

#### College Experience Survey

Participants were asked about their past research experience, either specifically in psychology or in general academia. Participants were asked to choose between three choices: *no research experience, intermediate research experience* (i.e., research work for class, research work for lab), and *advanced research experience* (i.e., professional research experience, published works).

#### Demographic and Background Questionnaire

Series of standard demographic questions were asked, including participants’ age, gender, and ethnicity.

## Results

### The Relationship Between Creativity and Critical Thinking

We performed a Pearson correlation to examine the relationship between creativity and critical thinking (the two-c), which include performances on three measures on creativity (*creativity originality*, *creativity fluency*, and *hypothesis generation*) and three measures on critical thinking (*experiment generation*, *CCT*, and *PCT*).

Most of the dependent variables had a significantly positive correlation. The only insignificant correlation was found between Sternberg hypothesis generation and CCT, *r*_(247)_=0.024, *p*=0.708 (see Table 1).

**Table 1:** Correlation coefficients for study variables.

Variable	N	1	2	3	4	5
1. Creativity fluency	210					
2. Creativity originality	197	0.484^**^				
3. Hypothesis generation	210	0.464^**^	0.355^**^			
4. Experiment generation	210	0.302^**^	0.274^**^	0.330^**^		
5. Psychological critical thinking	210	0.265^**^	0.259^**^	0.292^**^	0.367^**^	
6. Critical thinking test	210	0.153^*^	0.173^*^	0.024	0.347^**^	0.152^*^

* *p*<0.05.

** *p*<0.01.

**Figure 1:**
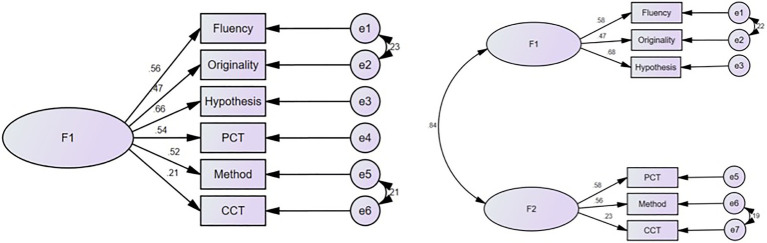
The comparison of the two confirmatory factor analysis (CFA) models: one-factor vs. two-factor.

Confirmatory factor analysis (CFA) was conducted by applying SEM through AMOS 21 software program and the maximum likelihood method. One-factor and two-factor models have been analyzed, respectively (see Figure 1).

As it is demonstrated in Table 2, the value ranges of the most addressed fit indices used in the analysis of SEM are presented. Comparing two models, *χ*^2^/df of the two-factor model is in a good fit, while the index of the one-factor model is in acceptable fit. The comparison of the two models suggest that the two-factor model is a better model than the one-factor model.

**Table 2:** Recommended values for evaluation and the obtained values.

Fit measure	Good fit	Acceptable fit	Obtained values	
			One-factor model	Two-factor model
*χ*^2^/df	0≤*χ*^2^/df≤2	2<*χ*^2^/df≤3	2.24	1.82
RMSEA	0≤RMSEA≤0.05	0.05<RMSEA≤0.08	0.05	0.04
NFI	0.95≤NFI≤1.00	0.90≤NFI<0.95	0.96	0.98
CFI	0.97≤CFI≤1.00	0.95≤CFI<0.97	0.98	0.99
GFI	0.95≤GFI≤1.00	0.90≤GFI<0.95	0.99	0.99
AGFI	0.90≤AGFI≤1.00	0.85≤AGFI<0.90	0.97	0.97

RMSEA,root mean square error of approximation; NFI, normed fit index; CFI, comparative fit index; GFI, goodness-of-fit index; and AGFI, adjusted goodness-of-fit-index (Schermelleh-Engel et al., 2003).

### Cross-Cultural Differences in Critical Thinking and Creativity

We conducted a 2 (Country: the United States vs. China)×2 (Two-C: Creativity and Critical Thinking) ANOVA to investigate the cultural differences in critical thinking and creativity. We averaged scores of three critical thinking measurement (*experiment generation*, *PCT*, and *CCT*) to represent critical thinking and averaged three creativity scores (*creativity originality*, *creativity fluency*, and *hypothesis generation*).

This analysis revealed a significant main effect for the type of thinking (i.e., creative vs. critical thinking), *F*_(1,247)_=464.77, *p*<0.01, *η*_p_^2^=0.653. Moreover, there was a significant interaction between country (i.e., the United States vs. China) and type of thinking, *F*_(1,247)_=62.00, *p*<0.01, *η*_p_^2^=0.201. More specifically, Chinese students (*M*=1.32, *SD*=0.59) outperformed American students (*M*=1.02, *SD*=0.44) on critical thinking. In contrast, American students (*M*=2.59, *SD*=1.07) outperformed Chinese students (*M*=2.05, *SD*=0.83) on creativity.

### Influence of Research Experience on Critical Thinking and Creativity

**Figure 2:**
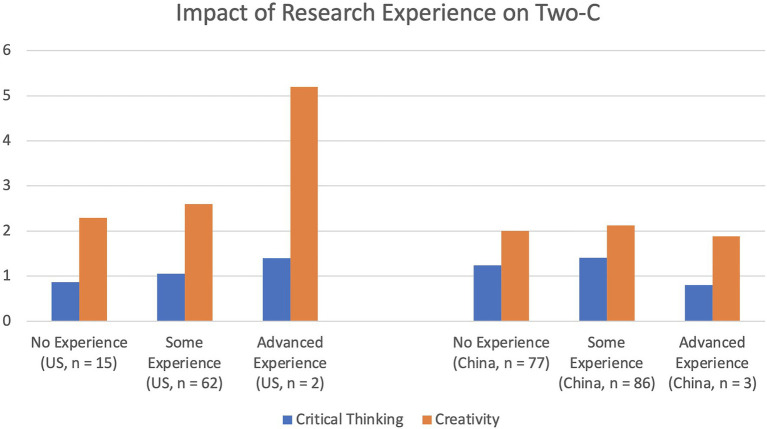
Estimated marginal means of Two-C for the United States and Chinese samples.

The last hypothesis states that having college research experience (through courses or research lab) would enhance students’ creativity and critical thinking from both countries. We performed a 2 (Two-C: Creativity and Critical Thinking)×2 (Country: the United States vs. China)×3 (Research Experience: Advanced vs. Some vs. No) ANOVA to test this hypothesis. This analysis revealed a significant main effect for research experience, *F*_(2,239)_=4.05, *p*=0.019, *η*_p_^2^=0.033. Moreover, there was a significant interaction between country (i.e., the United States vs. China) and research experience, *F*_(2,239)_=5.77, *p*=0.004, *η*_p_^2^=0.046. In addition, there was a three-way interaction among country, two-C, and research experience. More specifically, with an increase of research experience for American students, both critical thinking and creativity improved. In contrast, for Chinese students, the impact of research experience was not significant for creativity. However, some research experience positively impacted Chinese students’ critical thinking (see Figure 2).

## Discussion

The current study aimed to investigate the relationship between creativity and critical thinking, how culture influences creativity and critical thinking, and how college research experience affects creativity and critical thinking. Our results supported the first hypothesis regarding the positive correlation among all of the dependent variables. The mean correlation between the measures of creativity and critical thinking was 0.230. This result was in line with the findings from previous research (Gibson et al., 1968; Gadzella and Penland, 1995; Siburian et al., 2019; Akpur, 2020; Qiang et al., 2020). Moreover, our confirmatory factor analysis yielded similar results as analysis of Wechsler et al. (2018) and Akpur (2020) and provides more evidence of the relative independence between creativity and critical thinking. We found that at the latent variable level, the two skills are highly correlated to each other (*r*=0.84). In addition, we found that although the one-factor model was an acceptable fit, a two-factor model was a better fit for analysis. This result suggests that despite the correlation between creativity and critical thinking, the two skills should be studied as separate factors for an appropriate and comprehensive analysis.

The results of this study partially confirmed our second hypothesis and replicated the findings from past studies (Niu et al., 2007; Lun et al., 2010; Wong and Niu, 2013; Tang et al., 2015). As predicted, there was a significant main effect for culture in students’ performance for all six measures in the two-C analysis model. United States students performed better than Chinese students in all three creativity measures, and Chinese students performed better than United States students in all critical thinking measures. Given the diversity in the type of measures used in this study, the results suggest that United States and Chinese students’ performance aligns with the stereotype belief found in study of Wong and Niu (2013). The findings from the current study suggest that the stereotype belief observed in both United States and Chinese students (United States students generally perform better on creativity tasks, while Chinese students perform typically better on critical thinking tasks) is not entirely unfounded. Furthermore, the clear discrepancy in performance between United States and Chinese students provides more evidence to suggest that creativity and critical thinking are relatively autonomous skills. Although, a high correlation between these two skills was found in our study, the fact that students from two different cultures have two different development trajectories in critical thinking and creativity suggests that these two skills are relatively autonomous.

Lastly, the results also confirmed our third hypothesis, that is, college research experience did have a positive influence on students’ creativity and critical thinking. Compared to students with no research experience, students with some research experience performed significantly better in all measures of creativity and critical thinking. This finding is consistent with the previous literature (Mill et al., 1994; Penningroth et al., 2007; Stevens and Witkow, 2014; Stevens et al., 2016; Kuo et al., 2018). The result of our study suggests that college research experience is significant to enhance both creativity and critical thinking. As research experience becomes a more essential component of college education, our results suggest that it not only can add credential for applying to graduate school or help students learn skills specific to research, but also help students enhance both creativity and critical thinking. Furthermore, it is worth noting that this nature held true for both Chinese and American students. To our knowledge, this is a first investigation examining the role of research experience in both creativity and critical thinking cross-culturally.

In addition to the report of our findings, we would like to address some limitations of our study. First, we would like to note that this is a correlational and cross-sectional study. A positive correlation between research experience and the two dependent variables does not necessarily mean causation. Our results indeed indicate a positive correlation between research experience and the two-C variables; however, we are not sure of the nature of this relationship. It is plausible that students with higher creativity and critical thinking skills are more engaged in research as much as it is to argue in favor of a reversed directional relationship. Second, we would like to note the sample bias in our study. Majority of our participants were female, majoring in the social sciences and a relatively high number of participants chose not to report their gender. Third, we would like to note that our study did not measure all creativity and critical thinking dimensions, we discussed in the introduction. Instead, we focused on a few key dimensions of creativity and critical thinking. Our primary focus was on divergent thinking, convergent thinking, and scientific creativity as well as few key dimensions of critical thinking (evaluation, logical reasoning, and probability thinking), scientific critical thinking involved in problem solving and hypothesis testing. Moreover, our results do not show what specific components of research training are beneficial for the enhancement of creativity and critical thinking.

For future research, a longitudinal design involving a field experiment will help investigate how different research training components affect the development of creativity and critical thinking. In addition, a cross-cultural study can further examine how and why the students from different cultures differ from each other in the development of these two potentials. As such, it might shed some light on the role of culture in creativity and critical thinking.

## Conclusion and Implication

The result of our study provides few insights to the study of creativity and critical thinking. First, creativity and critical thinking are a different construct yet highly correlated. Second, whereas Americans perform better on creativity measures, Chinese perform better on critical thinking measures. Third, for both American and Chinese students, college research experience is a significant influence on the enhancement of creativity and critical thinking. As research experience becomes more and more essential to college education, its role can not only add professional and postgraduate credentials, but also help students enhance both creativity and critical thinking.

Based on our results, we recommend that research training be prioritized in higher education. Moreover, each culture has strengths to develop one skill over the other, hence, each culture could invest more in developing skills that were found to be weaker in our study. Eastern cultures can encourage more creativity and Western cultures can encourage more critical thinking.

To conclude, we would like to highlight that, although recognized globally as essential skills, methods to foster creativity and critical thinking skills and understanding creativity and critical thinking as a construct requires further research. Interestingly, our study found that experience of research itself can help enhance creativity and critical thinking. Our study also aimed to expand the knowledge of creativity and critical thinking literature through an investigation of the relationship of the two variables and how cultural background influences the performance of these two skills. We hope that our findings can provide insights for researchers and educators to find constructive methods to foster students’ essential 21st century skills, creativity and critical thinking, to ultimately enhance their global competence and life success.

## Data Availability Statement

The original contributions presented in the study are included in the article/supplementary material, further inquiries can be directed to the corresponding author.

## Ethics Statement

The studies involving human participants were reviewed and approved by Institutional Review Board at Pace University. The participants provided their informed consent online prior to participating in the study.

## Author Contributions

## All authors listed have made a substantial, direct, and intellectual contribution to the work, and approved it for publication.

## Conflict of Interest

The authors declare that the research was conducted in the absence of any commercial or financial relationships that could be construed as a potential conflict of interest.

## Publisher’s Note

All claims expressed in this article are solely those of the authors and do not necessarily represent those of their affiliated organizations, or those of the publisher, the editors and the reviewers. Any product that may be evaluated in this article, or claim that may be made by its manufacturer, is not guaranteed or endorsed by the publisher.

## Funding

This work was supported by the International Joint Research Project of Faculty of Education, Beijing Normal University (ICER201904), and a scholarly research funding by Pace University.
